# Supplementary material for: A pilot study of lymphodepletion intensity for peripheral blood mononuclear cell-derived neoantigen-specific CD8 + T cell therapy in patients with advanced solid tumors
Source: Nat Commun. 2023 Jun 10;14:3447. doi: 10.1038/s41467-023-39225-7 (PMC10257664; doi:10.1038/s41467-023-39225-7)
Supplement: Supplementary file 1 — Supplementary Information [file 41467_2023_39225_MOESM1_ESM.pdf]

Supplementary Tables and Figures

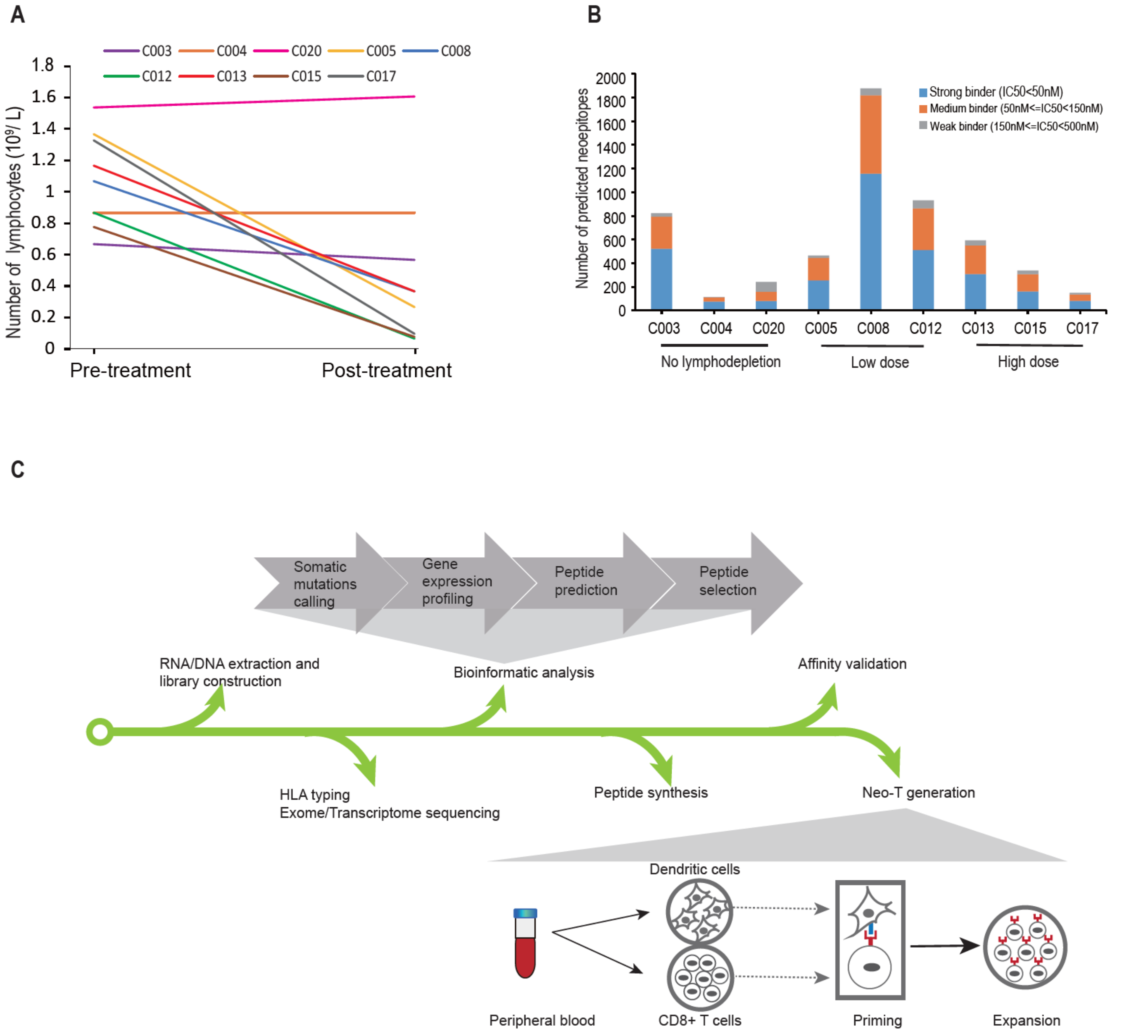

Figure S1. Features of patients and Neo-T manufacture pipeline. (A). Lymphocyte counts before and after lymphodepleting treatment. (B) Number and quality of predicted neoepitopes in each patient. (C). Schematic diagram of neoantigen prediction and Neo-T manufacturing pipeline (methods). Source data are provided as a Source Data file.

A

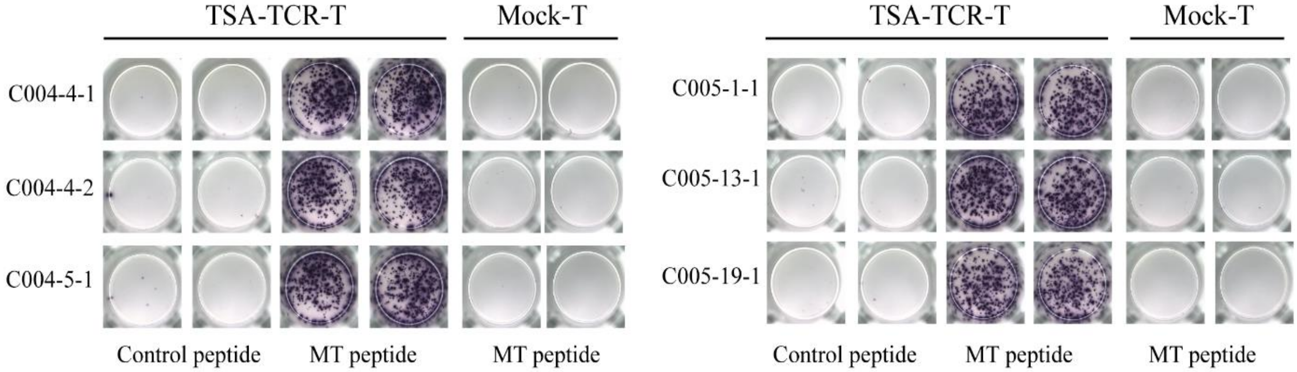

Figure S2. Neoantigen-specific T cells of Neo-T products from patient C004 and C005. (A). Functional validation of representative TSA-specific TCR clones from patient C004 and C005. IFN-γ secreting cells were detected by ELISPOT assay. TSA, tumor specific antigen. Source data are provided as a Source Data file.

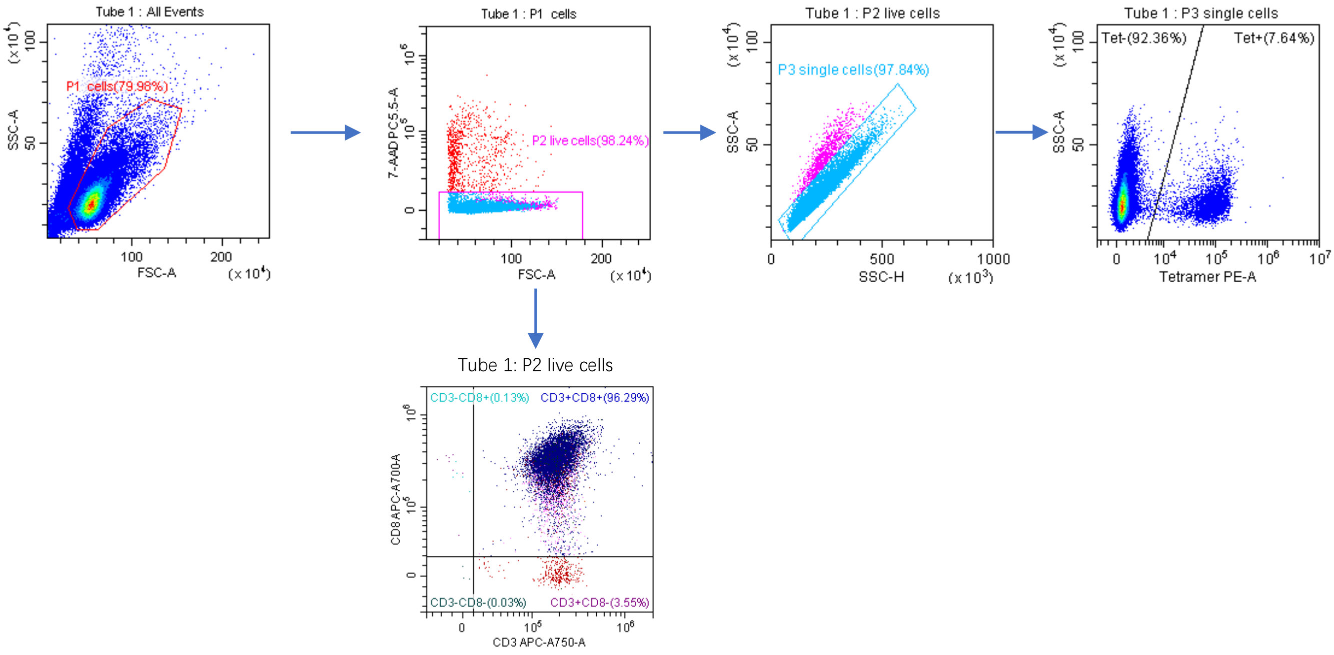

Figure S3. Representative images of flow cytometry analyses of Neo-Ts with gating. Results of CD8+T cell fraction were shown in Table s1, percentage of Tetramer+ cells were showed in Table S2. Source data are provided as a Source Data file.

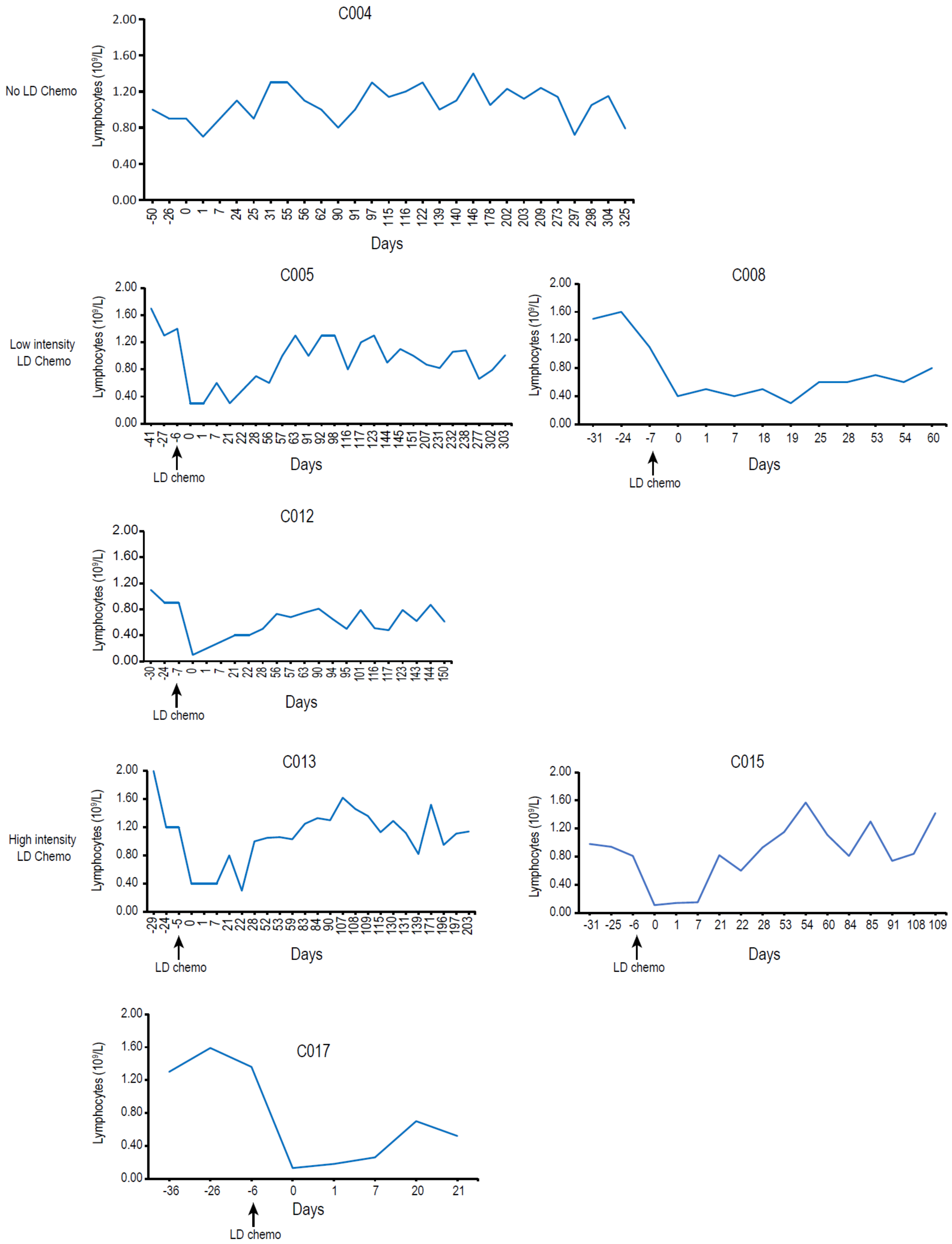

Figure S4: Total lymphocyte counts in different patients before and after lymphodepletion chemotherapy. Source data are provided as a Source Data file

Table S1. Characteristics of Neo-Ts Generated from All Patients

Patient No.	#Nons Mutations	#Neoantigens used for Neo-Ts	#Average number of Neo-Ts (*10^7)	Average Cell viability of Neo-Ts	Average fraction of CD8+T in Neo-Ts	Bacteria/fungus/mycoplasma test of Neo-Ts
C001	302	5	4.78	0.96	0.97	-/-/-
C003	5471	9	15.68	0.97	0.90	-/-/-
C004	838	13	20.41	0.97	0.97	-/-/-
C005	2311	11	9.67	0.97	0.96	-/-/-
C008	13024	8	20.23	0.98	0.93	-/-/-
C012	3425	11	10.37	0.99	0.98	-/-/-
C013	1921	15	15.80	0.96	0.98	-/-/-
C015	529	12	12.20	0.98	1.00	-/-/-
C017	47	8	33.00	0.98	0.97	-/-/-
C018	41	8	84	0.91	0.97	-/-/-
C020	179	20	90	0.88	0.99	-/-/-

Table S2. Ratio of Neoantigen specific T cells in Neo-Ts based on Tetramer analysis

**Patient#**		**003**		**004**		**005**		**008**		**012**		**013**		**015**		**017**		**020**
	#1	3.78%	#4	1.32%	#1	1.29%	#1	0.22%	#2	0.00%	#3	32.13%	#4	0.06%	#1	0.00%	#12	2.39%
	#3	0.79%	#5	12.99%	#3	0.05%	#7	6.92%	#3	0.48%	#4	0.00%	#9	0.11%	#2	0.00%		
	#5	0.64%	#14	4.51%	#7	1.06%	#13	5.42%	#4	0.78%	#6	0.00%	#10	0.07%	#9	0.00%		
	#8	0.24%	#16	0%	#15	0.18%	#14	2.07%	#6	0.00%	#8	1.25%	#11	0.04%	#10	0.01%		
			#18	0.45%	#16	1.49%	#15	4.77%	#11	0.41%	#10	0.00%	#13	0.00%	#11	0.00%		
					#19	0.45%	#16	1.14%	#19	0.00%	#12	0.00%	#15	0.11%	#19	0.00%		
neoantigens							#17	1.35%	#20	7.80%	#14	0.00%	#16	0.01%	#20	16.12%		
							#18	0.36%	#23	15.80%	#19	0.00%	#20	0.01%				
							#19	5.02%			#22	10.53%	#22	0.00%				
							#20	1.34%										
							#22	4.78%										
Total:		5.44%		19.27%		4.52%		33.40%		25.27%		43.92%		0.41%		16.13%		2.39%

Table S3. List of Neoantigens Tested from patient C004 and C005.

Peptide ID	Patient ID	Gene ID	Mutation types	Protein change	Length (aa)	HLA allele (HLA-)	Mutated peptide		Wiletype peptide		Gene expression (TPM)	Frequency in tumor	Affinity through T2 assay (FI)
							Sequence	Affinity (nM)	Sequence	Affinity (nM)			
C004-1	C004	RADIL	SNV	p.A689T	9	A1101	ATGEHFFQK	7.9	AAGEHFFQK	28.5	1.68	0.05	0.40
C004-2	C004	NNT	SNV	p.C44Y	9	A1101	YTHQELWYK	13.8	YTHQELWCK	27.5	49.80	0.05	0.27
C004-3	C004	SLC25A46	INDEL	-	9	A1101	LAFWSVLKK	14.6	-	-	74.64	0.02	0.25
C004-4	C004	TTN	INDEL	-	9	A1101	SAWYTAINK	17.0	-	-	9.10	0.02	2.44
C004-5	C004	SLC25A46	INDEL	-	9	A1101	ILAFWSVLK	17.0	-	-	74.64	0.02	18.69
C004-9	C004	TCF25	SNV	p.V276M	9	A1101	MVLLQTSPY	34.9	VVLLQTSPY	51.5	44.61	0.07	0.55
C004-11	C004	SUSD2	SNV	p.R250W	9	A1101	GALWIIDSK	46.0	GALRIIDSK	74.3	12.51	0.05	0.46
C004-14	C004	TGFBRAP1	SNV	p.H725R	9	A1101	HTLLAIYLR	84.2	HTLLAIYLH	50000.0	34.17	0.05	19.89
C004-17	C004	GSTZ1	SNV	p.E32K	9	A1101	KTVPINLIK	12.2	ETVPINLIK	63.4	16.11	0.04	1.44
C004-18	C004	ZZEF1	SNV	p.D845A	9	A1101	ASVPMEILK	12.4	DSVPMEILK	50000.0	27.76	0.04	0.94
C004-21	C004	AKAP6	SNV	p.Q762H	9	A1101	ATKSALIHK	15.5	ATKSALIQK	16.8	5.29	0.05	4.69
C004-22	C004	NPAT	SNV	p.V575I	9	A1101	SSDSSEIHK	18.6	SSDSSEVHK	29.5	40.81	0.04	0.63
C004-23	C004	IGBP1	INDEL	-	9	A1101	AAGSRTLYK	19.3	-	-	34.90	0.04	1.40
C005-1	C005	PAQR6	SNV	p.P92L	9	A0201	FLLPACLYL	3.6	FLLPACLYP	56.0	4.92	0.10	1.44
C005-3	C005	ATG9A	SNV	p.A124V	9	A0201	FLPAQVCSV	4.2	FLPAQVCSA	15.2	19.99	0.17	1.38
C005-7	C005	NCAPG2	SNV	p.P388S	9	A0201	SLLEDSYPM	5.1	SLLEDPYPM	5.9	24.43	0.14	0.73
C005-12	C005	ST5	SNV	p.G1024E	9	A0201	TLNELVSEV	10.4	TLNGLVSEV	13.9	52.33	0.21	1.58
C005-13	C005	TXNIP	SNV	p.G266D	9	A0201	ILDCNILRV	10.5	ILGCNILRV	16.0	364.07	0.08	2.73
C005-14	C005	*KDM4A*	SNV	p.P870S	9	A0201	MQSDDWPFV	11.4	MQPDDWPFV	22.0	23.87	0.06	1.15
C005-15	C005	*IDH3B*	SNV	p.E124K	9	A0201	KLASYDMRL	12.1	ELASYDMRL	164.3	28.88	0.05	0.79
C005-16	C005	*ERBIN*	SNV	p.S274L	9	A0201	GLLKNITTL	12.7	GSLKNITTL	50000.0	98.36	0.05	1.59
C005-19	C005	*ZSWIM8*	SNV	p.A85T	9	A0201	VTFHIPFEV	15.5	VAFHIPFEV	41.1	20.10	0.06	1.23
C005-25	C005	*SPIN3*	SNV	p.P156S	9	A0201	SVMNTWFYI	11.5	PVMNTWFYI	50000.0	10.66	0.10	2.96
C005-29	C005	*ANOS1*	SNV	p.P613L	9	A0201	ILLSDHYVL	19.0	ILPSDHYVL	51.3	7.42	0.33	2.79

Supplementary Note

Clinical Study Protocol Synopsis

Title	Phase I Clinical Trial of TSA-CTL (Tumor Specific Antigen-Induced Cytotoxic T Lymphocytes) In the Treatment of Advanced Solid Tumors
Enrollment	24
Site	Sun Yat-sen University Cancer Center
Study Phase	Phase I (Investigator-initiated trial)
Study Objectives	1.Primary objective: to evaluate the safety of TSA-CTL in the treatment of advanced solid tumors.<br>2.Secondary objective: to evaluate preliminarily the efficacy of TSA-CTL in the treatment of advanced solid tumors.
Conditions	Advanced solid tumors
Study Drug	TSA-CTL (Tumor Specific Antigen-Induced Cytotoxic T Lymphocytes)
Dose and Method of Administration	$5x10^7$~$1.2x10^9$ cells，intravenous infusion
Study Design	This is a single arm, open label and non-randomized clinical study with two parts.<br>In Part 1, 9 subjects with advanced solid tumors will be enrolled into Groups A (no non-myeloablative lymphodepletion), B and C (non-myeloablative lymphodepletion with different chemotherapy intensities) to assess the safety and dose intensity of non-myeloablative lymphodepletion chemotherapy before cell infusion. In Group A, subjects No. 1, 2 and 3 will receive 6 infusions of $5x10^7$, $1x10^8$, and $2x10^8$ cells, respectively. Treatment of subject No. 2 will begin after safety evaluation of subject No.1. Doses of TSA-CTL cell infusions in subsequent subjects will be determined after safety evaluation of subjects No. 1-3.<br>Depending on results in Part 1, the study may proceed to Part 2, where 15 subjects with advanced solid tumors will be enrolled to receive TSA-CTL cell infusions with or without non-myeloablative lymphodepletion.<br>Group A:<br>No lymphodepletion. Patients will receive TSA-CTL iv over 20-30 minutes on day 0.<br>Group B:<br>Patients will receive a low-dose lymphodepletion regimen consisting of cyclophosphamide and fludarabine followed by TSA-CTL.<br>TSA-CTL: iv over 20-30 minutes on day 0.<br>Cyclophosphamide: 500 $mg/m^{2/}$day iv on day -5 for one day.<br>Fludarabine: 25 mg/m^2/day iv over 30 minutes on day -5 and -4 for two days.<br>Group C:<br>Patients will receive a medium-dose lymphodepletion regimen consisting of cyclophosphamide and fludarabine followed by TSA-CTL.<br>TSA-CTL: iv over 20-30 minutes on day 0.<br>Cyclophosphamide: 500 $mg/m^{2/}$day iv on day -5 and -4 for two days.<br>Fludarabine: 25 mg/m^2/day iv over 30 minutes on day -5 and -4 for two days.

Inclusion Criteria	1. Greater than or equal to 18 years of age and less than or equal to 70 years of age; all genders.<br>2. Advanced solid tumors including but not limited to some high frequency somatic mutations, such as melanoma, colorectal cancer, gastric cancer, esophageal cancer, squamous cell carcinoma of the lung, triple-negative breast cancer, etc.<br>3. Advanced solid tumors patients who are HLA - A0201 /A1101/A2402 subtypes.<br>4. Measurable solid tumors with at least one lesion that is resectable or tumor biopsies for DNA extraction.<br>5. Patients who failed or were intolerant to standard treatment.<br>6. Patients (or their legal representatives) who are able to understand and sign the Informed Consent Form and willing to sign a durable power of attorney.<br>7. Clinical performance status of ECOG is 0 or 1 and expected lifetime is greater than six month and patients who are able to cooperate to observe adverse reactions and the effect of the treatment.<br>8. Patients of both genders must be willing to practice birth control from the time of enrollment to five months after treatment on this study.<br>9. Serology: HIV antibody(-), hepatitis B antigen(-), and hepatitis C antibody(-). A fertile woman must have a negative pregnancy test. Hematology: Absolute neutrophil count is greater than 1500/mm^3 without the support of filgrastim; WBC is greater than or equal to 3000/mm^3; lymphocyte count is greater than or equal to 800/mm^3; Platelet count is greater than or equal to 100,000/mm^3; Hemoglobin is greater than or equal to 9.0 g/dL ; Chemistry: Serum ALT/AST is less than or equal to 2.5 times the upper limit of normal; Serum Creatinine is less than or equal to 1.5 times the upper limit of normal ; Total bilirubin is less than or equal to 1.5 the upper limit of normal, except in patients with Gilbert's Syndrome who must have a total bilirubin less than 3 times the upper limit of normal.<br>10. More than four weeks must have elapsed since any prior systemic therapy at the time the patient receives the lymphodepletion regimen, and toxicities must have recovered to grade 1 or less (except for toxicities such as alopecia or vitiligo).<br>Note: Patients may have undergone minor surgical procedures within the past 3 weeks, as long as all toxicities have recovered to grade 1 or less.

Exclusion Criteria	1. Pregnant or lactating women.<br>2. Any primary immunodeficiency (such as Severe Combined Immunodeficiency Disease).<br>3. Opportunistic infection<br>4. History of autoimmune disease.<br>5. Active systemic infections, coagulation disorders or other active major medical illnesses of the cardiovascular, respiratory or immune system.<br>6. Systemic steroid therapy in the past 4 weeks.<br>7. History of severe immediate hypersensitivity reaction to any of the agents used in this study.<br>8. Patients with unstable brain metastases.<br>9. Choroidal melanoma and clear cell sarcoma patients.<br>10. Negative for expression of MHC molecules.
Outcome Measures	Primary Outcome Measure: number of participants with adverse events, as assessed by CTCAE v5.0, within 30 days after the first infusion.<br>Secondary Outcome Measures:<br>1.Disease Control Rate (DCR): proportion of participants with tumor size reduction (CR, PR) and stable disease (SD) assessed by RECIST 1.1 and iRECIST.<br>2. Overall survival (OS): time from the first infusion of Investigational Product until death.<br>3. Progression-free survival (PFS): time from the first infusion of Investigational Product until objective tumor progression, as assessed by RECIST 1.1 and iRECIST, or death, whichever occurs first.<br>4. Duration of Response (DOR) refers to the period from the first evaluation of tumor as CR or PR to the first evaluation as PD (Progressive Disease) per RECIST1.1 and iRECIST.
Statistic analysis plan	**<u>Analysis datasets</u>**<br>Full Analysis Set (FAS): All subjects who sign an informed consent form, are enrolled and receive at least one dose of study treatment.<br>Per-Protocol Set (PPS): Subjects who receive at least one dose of study treatment in FAS and have at least one post-baseline tumor assessment. PPS is a secondary efficacy anaslysis dataset.<br>Safety Set (SS): All subjects who are enrolled and receive at least one dose of study treatment.<br>**<u>Efficacy analysis</u>**<br>Efficacy analysis will be based on FAS. PPS analysis is secondary to FAS. Objective tumor imaging data will be evaluated by investigators according to RECIST1.1 (main analysis) and iRECIST.<br>**<u>Safety analysis</u>**<br>Safety analysis is based on SS, ie, a descriptive analysis of safety data among treated subjects in each cohort/treatment group and overall.<br>Descriptive summary statistics (eg, mean, standard deviation, median, minimum and maximum) of laboratory findings, vital signs and ECG parameters, and changes from baseline.

	Only treatment-emergent adverse events (TEAEs) will be pooled and analyzed. TEAEs will be statistically described, includng the number and percentage of subjects, based on SS in each cohort/treatment group and overall. All adverse events will be listed in the data table. The incidence of TEAEs will be summarized by SOC, PT, CTCAE grade, and relationship with study treatment.
