# Supplementary material for: A pilot study of lymphodepletion intensity for peripheral blood mononuclear cell-derived neoantigen-specific CD8 + T cell therapy in patients with advanced solid tumors
Source: Nat Commun. 2023 Jun 10;14:3447. doi: 10.1038/s41467-023-39225-7 (PMC10257664; doi:10.1038/s41467-023-39225-7)
Supplement: Supplementary file 3 — Reporting Summary [file 41467_2023_39225_MOESM3_ESM.pdf]

nature portfolio

Corresponding author(s): Xiaoshi Zhang, Xi Zhang, Rui-Hua Xu

Last updated by author(s): May 30, 2023

Reporting Summary

Nature Portfolio wishes to improve the reproducibility of the work that we publish. This form provides structure for consistency and transparency in reporting. For further information on Nature Portfolio policies, see our Editorial Policies and the Editorial Policy Checklist.

Statistics

For all statistical analyses, confirm that the following items are present in the figure legend, table legend, main text, or Methods section.

n/a	Confirmed	
☐	☒	The exact sample size (*n*) for each experimental group/condition, given as a discrete number and unit of measurement
☐	☒	A statement on whether measurements were taken from distinct samples or whether the same sample was measured repeatedly
☐	☒	The statistical test(s) used AND whether they are one- or two-sided<br>*Only common tests should be described solely by name; describe more complex techniques in the Methods section.*
☒	☐	A description of all covariates tested
☒	☐	A description of any assumptions or corrections, such as tests of normality and adjustment for multiple comparisons
☒	☐	A full description of the statistical parameters including central tendency (e.g. means) or other basic estimates (e.g. regression coefficient) AND variation (e.g. standard deviation) or associated estimates of uncertainty (e.g. confidence intervals)
☒	☐	For null hypothesis testing, the test statistic (e.g. *F*, *t*, *r*) with confidence intervals, effect sizes, degrees of freedom and *P* value noted<br>*Give P values as exact values whenever suitable.*
☒	☐	For Bayesian analysis, information on the choice of priors and Markov chain Monte Carlo settings
☒	☐	For hierarchical and complex designs, identification of the appropriate level for tests and full reporting of outcomes
☒	☐	Estimates of effect sizes (e.g. Cohen's *d*, Pearson's *r*), indicating how they were calculated

Our web collection on statistics for biologists contains articles on many of the points above.

Software and code

Policy information about availability of computer code

Data collection	DNA and RNA were extracted from FFPE tumor tissues using RecoverAll™ Total Nucleic Acid Isolation Kit (Invitrogen, Cat#:AM1975), libraries construction, and sequencing (pair-end 150bp) were conducted according to protocols on Illumina platform. For TCR-seq, VDJ and 5' transcriptome libraries of CD8+tetramer+ cells were constructed and sequenced on HiSeq X instruments (Illumina) platform (pair-end 150bp).
Data analysis	For sequencing data, low-quality reads were removed with SOAP nuke v1.5. Whole-exome sequencing data were aligned to reference genome (hg19) using DRAGEN pipeline. Furthermore, indel-realignment and recalibration were performed by the Genome Analysis Toolkit (GATK). MuTect and Strelka were used to identify SNVs and InDels, respectively. RNA sequencing reads were mapped to reference genome through STAR (version 2.5.3a). Gene expression (Transcripts Per Million, TPM) was calculated by using RSEM.<br>For TCR-seq analysis, raw data processing was performed using the Cell Ranger version 2.1 pipeline (10x Genomics). The 'vdj' and 'count' of Cell Ranger were used to analyze the expression of gene and identify TCRs, respectively. TCRs were further compared with the PIRD database (https://db.cngb.org/pird/tbadb/) to remove the pathogen-specific TCRs, and the obtained mutant epitope-specific TCRs.<br>Affinity of each mutant peptides and corresponding HLA alleles from same patient was predicted by NetMHC 3.0, NetMHCpan 4.0, PickPocket, PSSMHCpan, and SMM.

For manuscripts utilizing custom algorithms or software that are central to the research but not yet described in published literature, software must be made available to editors and reviewers. We strongly encourage code deposition in a community repository (e.g. GitHub). See the Nature Portfolio guidelines for submitting code & software for further information.

Data

Policy information about availability of data

All manuscripts must include a data availability statement. This statement should provide the following information, where applicable:

- Accession codes, unique identifiers, or web links for publicly available datasets
- A description of any restrictions on data availability
- For clinical datasets or third party data, please ensure that the statement adheres to our policy

The reference genome (hg19) can be accessed at (http://hgdownload.cse.ucsc.edu/goldenpath/hg19/chromosomes/). PIRD database is accessible at (https://db.cngb.org/pird/tbadb/). The sequencing data generated in this study can be accessed through GSA under the accession code HRA004715 (https://ngdc.cncb.ac.cn/gsa-human/browse/HRA004715). Sequencing data are available under restricted access. Access can be obtained by completing the application form via GSAHuman System and/or by contacting the corresponding authors. The clinical Study Protocol Synopsis is available as Supplementary Note in the Supplementary Information file. Other individual deidentified participant data will be shared upon request from the corresponding authors. The remaining data are available within the Article, Supplementary Information or Source Data file.

Human research participants

Policy information about studies involving human research participants and Sex and Gender in Research.

Reporting on sex and gender	This phase I clinical trial recruited cancer patients from both genders, and gender was determined based on self-reporting. There were 8 males and 3 females enrolled in this phase I trial.
Population characteristics	Eleven patients (median age 51 years; range 29-66 years) with locally advanced or metastatic cancer were enrolled, including 9 patients with melanoma, 1 with colorectal cancer, and 1 with intrahepatic cholangiocarcinoma.
Recruitment	Eleven patients with advanced cancer (stage IV) were recruited for the study. Eligible patients should satisfy the following conditions: (1) 18 to 70 years of age with a pathologically confirmed diagnosis of metastatic or locally advanced cancer; (2) progressed after receiving at least one standard first-line treatment; (3) have an Eastern Cooperative Oncology Group (ECOG) performance status of no greater than 1; (4) HIV antibody negative, Treponema pallidum negative, Hepatitis C virus antibody negative, and HBV DNA negative; (5) HLA type is HLA-A11:01+ or HLA-A02:01+.<br>Patients were assigned to three treatment arms according to the time of enrollment. First three patients started with no LD chemo, next three patients with low LD chemo and the following three enrolled treated with high LD chemo
Ethics oversight	Institutional Ethics Committee at the Sun Yat-sen University Cancer Center

Note that full information on the approval of the study protocol must also be provided in the manuscript.

Field-specific reporting

Please select the one below that is the best fit for your research. If you are not sure, read the appropriate sections before making your selection.

[x] Life sciences [ ] Behavioural & social sciences [ ] Ecological, evolutionary & environmental sciences

For a reference copy of the document with all sections, see nature.com/documents/nr-reporting-summary-flat.pdf

Life sciences study design

All studies must disclose on these points even when the disclosure is negative.

Sample size	Eleven patients participated in this 3+3 dose-escalation Phase I clinical trial, with three patients in each dose level group, and two patients withdrew early from the trial.
Data exclusions	No data were excluded.
Replication	Lab experiments (ELISA) have 2 replicates with close results.
Randomization	Patients were randomly assigned to three treatment groups, with three patients in each dose level group.
Blinding	This is a phase I clinical trial to evaluate the safety of lymphodepletion during adoptive T cell therapy with Neo-T. This is a dose-escalation trial, so the investigators were not blinded to group allocation.

Reporting for specific materials, systems and methods

We require information from authors about some types of materials, experimental systems and methods used in many studies. Here, indicate whether each material, system or method listed is relevant to your study. If you are not sure if a list item applies to your research, read the appropriate section before selecting a response.

Materials & experimental systems

n/a	Involved in the study
☐	☒ Antibodies
☒	☐ Eukaryotic cell lines
☒	☐ Palaeontology and archaeology
☒	☐ Animals and other organisms
☐	☒ Clinical data
☒	☐ Dual use research of concern

Methods

n/a	Involved in the study
☒	☐ ChIP-seq
☐	☒ Flow cytometry
☒	☐ MRI-based neuroimaging

Antibodies

Antibodies used	APC-Cy™7 Mouse Anti-Human CD3 (BD Biosciences, 557832), Alexa Fluor® 700 Mouse Anti-Human CD8 (BD Biosciences, 557945),
Validation	All antibodies were commercially available and validated by the manufacturers.

Clinical data

Policy information about clinical studies

All manuscripts should comply with the ICMJEguidelines for publication of clinical research and a completedCONSORT checklist must be included with all submissions.

Clinical trial registration	NCT02959905
Study protocol	Study protocol can be find at: https://clinicaltrials.gov/ct2/show/NCT02959905
Data collection	Nine patients were enrolled at the Sun Yat-sen University Cancer Center from Feb.10, 2017 to Jun.19, 2019, and CT scan were performed at the hospital every two months for one year after T cell infusion.
Outcomes	The primary end point was safety and the secondary end point was objective response rate (ORR). In the safety assessment, adverse events were categorized and graded according to Common Terminology Criteria for Adverse Events (CTCAE) Version 5.0. CT (Computed Tomography) was conducted every two months after first cell infusion. Clinical efficacy was evaluated according to Response Evaluation Criteria in Solid Tumors (RECIST) 1.1.

Flow Cytometry

Plots

Confirm that:

☒ The axis labels state the marker and fluorochrome used (e.g. CD4-FITC).

☒ The axis scales are clearly visible. Include numbers along axes only for bottom left plot of group (a 'group' is an analysis of identical markers).

☒ All plots are contour plots with outliers or pseudocolor plots.

☒ A numerical value for number of cells or percentage (with statistics) is provided.

Methodology

Sample preparation	For neoantigen specific T cell analysis, CD8+ T cells isolated from PBMC of cancer patients were expanded in vitro for 25 days in the presence of mutant peptides and IL-2. After resting in cytokine-free culture medium overnight, the cells were re-stimulated with mutant peptides for 20 h. Then the T cells were co-stained with CD8, and individual peptide bound MHC-tetramers and analyzed by FACS.
Instrument	BD FACSAria II, Beckman CytoFLEX, Beckman DxFLEX
Software	FlowJo version 10
Cell population abundance	No sorting were performed.
Gating strategy	Every flow cytometry analysis was initiated as follows:<br>FSC-A/SSC-A to gate the lymphocyte population,<br>Gating of the Propidium Iodide or 7-AAD negative cells to select live cells, then analysis of CD8+% cell in live cells, meanwhile, gating on single cells in live cells and then analysis of MHC tetramer+% cells in single cells.

☒ Tick this box to confirm that a figure exemplifying the gating strategy is provided in the Supplementary Information.
